# Enhanced differentiation between 3‐hydroxyglutaric and 2‐hydroxyglutaric acids facilitates diagnostic testing for glutaric aciduria type 1

**DOI:** 10.1002/jmd2.12447

**Published:** 2024-08-27

**Authors:** Denis Cyr, Michel Boutin, Bruno Maranda, Paula J. Waters

**Affiliations:** ^1^ Medical Genetics Service, Department of Laboratory Medicine University of Sherbrooke Hospital Centre (CHUS) Sherbrooke Quebec Canada; ^2^ Department of Pediatrics University of Sherbrooke Sherbrooke Quebec Canada

**Keywords:** 2‐hydroxyglutaric acid, 3‐hydroxyglutaric acid, gas chromatography–tandem mass spectrometry, glutaric acid, glutaric acidemia type 1, glutaric aciduria type 1

## Abstract

Glutaric aciduria type 1 (GA1) is an inherited neurometabolic disorder, in which deficiency of glutaryl‐CoA dehydrogenase leads to accumulation of glutaric acid (GA) and 3‐hydroxyglutaric acid (3‐HG). Some low excretors may exhibit only slight elevation of urinary 3‐HG, with normal urinary GA, yet are at significant risk of severe clinical disease. Accurate quantitation of urinary 3‐HG is crucial in diagnostic workup for GA1, but in this context, current gas chromatography–mass spectrometry (GC–MS) methods have inherent analytical challenges. Co‐elution and spectral similarities of the 3‐HG and 2‐HG structural isomers can cause difficulties in quantitation of slightly elevated 3‐HG. Our laboratory recently acquired a gas chromatography system coupled to a triple quadrupole mass spectrometer (GC–MS/MS), and we took advantage of its increased sensitivity and specificity to improve our existing GC–MS method. A stable isotope dilution process is used, with sample treatment consisting of a double liquid–liquid extraction followed by a trimethylsilyl derivatization. The transitions *m*/*z* 349 → 333 for 3‐HG and *m*/*z* 349 → 321 for 2‐HG were selected to differentiate these two isobaric molecules based on their characteristic fragments, thus minimizing interferences despite co‐elution. Method validation demonstrated satisfactory precision and accuracy. Using GC–MS/MS instead of GC–MS allowed us to decrease the required specimen volume, number of sample processing steps, chromatographic run time, and instrument maintenance. This enhanced assay facilitates clinical laboratory testing for GA1, both in confirmatory protocols following positive newborn screening and in diagnostic investigation of patients with suggestive signs or symptoms.

**Synopsis**

Coupling tandem mass spectrometry to gas chromatography allows discrimination and quantitation of 3‐hydroxyglutaric and 2‐hydroxyglutaric acids by the analysis of their characteristic fragments.

## 1 INTRODUCTION

Glutaric aciduria type 1 (GA1; OMIM #231670) is an autosomal recessive metabolic disorder of lysine metabolism with an estimated worldwide incidence of 1:90 000–1:120 000^1^ and is characterized by severe neurological deterioration in infancy.^1^, ^2^ This rare inborn error of metabolism is caused by pathogenic variants in the *GCDH* gene, which encodes glutaryl‐CoA dehydrogenase. Loss of GCDH enzymatic activity leads to accumulation of glutaric acid (GA), 3‐hydroxyglutaric acid (3‐HG), glutaconic acid and glutarylcarnitine in body fluids.^1^ As GA1 is a treatable disease, and can be screened for by tandem mass spectrometry (MS–MS) analysis of acylcarnitines in dried bloodspots, many countries have incorporated this condition into their newborn screening (NBS) panels.^1^, ^3^ Upon diagnosis, dietary treatment including restriction of the amino acid precursors of glutaryl‐CoA, and l‐carnitine supplementation, can rapidly be initiated in order to reduce the risk of serious complications.^4^

A positive NBS result for GA1 triggers further biochemical investigations, usually including analysis of an organic acid profile in urine by gas chromatography–mass spectrometry (GC–MS)^5^, ^6^ and/or quantitation of GA and 3‐HG by a more precise stable isotope dilution GC–MS method.^7^ Notably, a considerable proportion of individuals with GA1 are “low excretors,” some of whom excrete normal levels of GA. However, an isolated increase of 3‐HG is strongly suggestive of GA1, therefore quantitation of 3‐HG is a cornerstone of diagnostic investigation for this disease, whether following NBS or prompted by suspicious clinical signs or symptoms. 3‐HG is thought to be generated by a specific intramitochondrial degradation of glutaryl‐CoA, its formation being quite stable and independent of the urinary GA concentration.^8^ Since 3‐HG is considered the major neurotoxin in this disease,^9^ it becomes very important to have a specific and sensitive method to quantitate this biomarker.

It is well known that chromatographic co‐elution of 2‐hydroxyglutaric acid (2‐HG) and 3‐HG isomers in routine GC–MS assays can cause analytical difficulty in unambiguously identifying mild elevations in urinary 3‐HG.^10^, ^11^ Increased 2‐HG excretion does not occur in GA1, but is a characteristic finding in several other neurometabolic disorders collectively known as 2‐hydroxyglutaric acidurias.^12^ Elevated 2‐HG excretion can also result from disorders of riboflavin metabolism, notably including multiple acyl‐CoA dehydrogenase deficiency (MADD), also known as glutaric aciduria type 2.^13^

Our analyses were performed using a GC interfaced to a triple quadrupole mass spectrometer (gas chromatography‐tandem mass spectrometry; GC–MS/MS). By selecting a precursor–fragment ion transition specific for each target compound, we were able to improve the sensitivity and specificity compared to our previous GC–MS method. To the best of our knowledge, this is the first report of a GC–MS/MS methodology using stable isotope dilution that has been validated and demonstrated capable of differentiating and quantitating the 2‐HG and 3‐HG isomers.

## 2 MATERIALS AND METHODS

### 2.1 Chemicals and reagents

3‐Hydroxyglutaric acid was obtained from Toronto Research Chemicals (North York, Canada). Glutaric and 2‐hydroxyglutaric acids were purchased from Sigma–Aldrich (St. Louis, MO). 3‐Hydroxyglutaric‐d_5_ and glutaric‐d_4_ acids (internal standards) were obtained from CND Isotopes (Pointe‐Claire, Canada). In all cases, we used the highest grade commercially available. *N*,*O*‐bis‐trimethylsilyl‐trifluoroacetamide (BSTFA +10% Trimethylchlorosilane [TMCS]) was from Regis Technologies (Morton Grove, IL) and ACS grade ethyl acetate from Fisher Scientific (Ottawa, Canada). Stock solutions of GA (5 mM), 2‐HG (2 mM), and 3‐HG (0.5 mM) were prepared in water and a working mixture containing 400 μM each of GA and 2‐HG, and 100 μM of 3‐HG, was obtained from stock solutions by dilution. This mixture, corresponding to the highest calibration point, was aliquoted and stored at −20°C. A stock solution of GA‐d_4_ (0.2 mM) and 3‐HG‐d_5_ (0.8 mM), the stable isotope‐labeled internal standard mixture, was prepared in water and stored at −20°C.

### 2.2 Sample extraction and derivatization

A 6‐point calibration, ranging from 2.5 to 400 μM for both GA and 2‐HG and from 1 to 100 μM for 3‐HG, was prepared daily by appropriate dilution of the working mixture solution. To 0.5 mL of each standard, urine sample, or quality control specimen, 50 μL of the internal standards solution was added and pH was adjusted to approximately 1 by adding 40 μL of 5 N HCl to each tube saturated with 0.1 g of sodium chloride. The sample was then extracted twice in succession with 2 mL of ethyl acetate, with vigorous shaking. The organic layers were combined into a second tube and the solvent was evaporated to dryness under a gentle stream of nitrogen. The residue was then derivatized by adding 100 μL of BSTFA +10% TMCS and heating for 60 min at 70°C.^5^ One microliter of each sample was injected into the GC–MS/MS system. Urinary creatinine concentration is determined separately by an enzymatic method in our core clinical biochemistry laboratory, using a Roche Cobas® Pro unit.

### 2.3 Gas chromatography and tandem mass spectrometry conditions

Method improvement was performed using an Agilent Technologies 7010B Triple Quadrupole GC–MS/MS equipped with a high‐efficiency electron impact (EI) ionization source. Chromatographic separation was achieved with two Phenomenex Zebron ZB‐5MSi columns (length, 15 m; internal diameter, 0.25 mm; film thickness, 0.25 μm) installed in tandem. This set‐up allows a backflush of the first column during the run, avoiding contamination of the second column and of the detector. The helium carrier‐gas flow rate was 1.2 mL/min in column 1 and 1.0 mL/min in column 2. The GC temperature program was as follows: initial temperature was 80°C, held for 1 min, then increased to 125°C at a rate of 6°C/min and held for 3 min. Next, it was raised to 170°C at a rate of 10°C/min, then increased to 300°C at a rate of 70°C/min and finally held for 5 min; resulting in a total run time of 23 min. A split injection mode (split ratio = 100:1) was used and 1 μL was injected at 250°C. Transfer line temperature was 280°C and ion source temperature was 230°C. In Multiple Reaction Monitoring (MRM) mode, nitrogen and helium were used as collision gas and quench gas, respectively. MRM is used for the MS recording of the ion transitions, *m*/*z* 349 → 333 for 3‐HG (qualifier *m*/*z* 349 → 185), *m*/*z* 349 → 321 for 2‐HG (qualifier *m*/*z* 349 → 203), *m*/*z* 261 → 147 for GA (qualifier *m*/*z* 261 → 233), and for the internal standards, 3‐HG‐d_5_ *m*/*z* 354 → 147 and GA‐d_4_ *m*/*z* 265 → 147.

## 3 RESULTS

**FIGURE 1:**
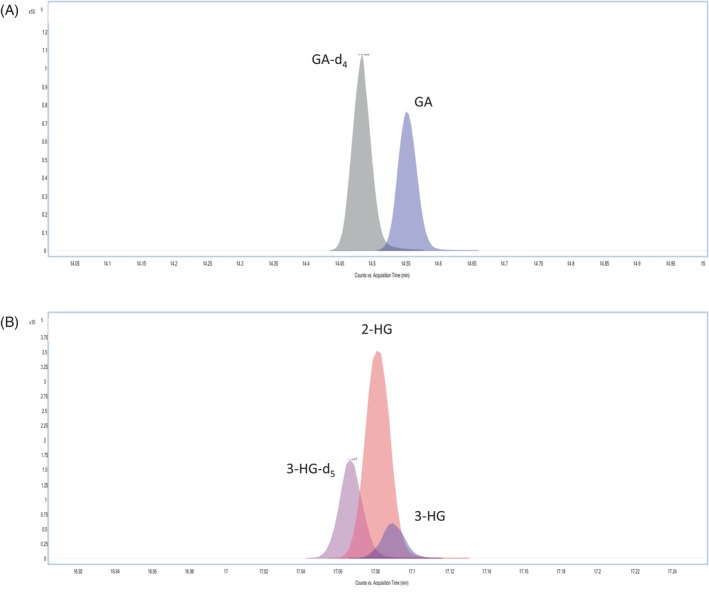
Chromatographic separation obtained using this GC–MS/MS method. (A) GA and GA‐d_4_. (B) 2‐HG, 3‐HG, and 3‐HG‐d_5_; showing the partial co‐elution of 2‐HG and 3‐HG.

**FIGURE 2:**
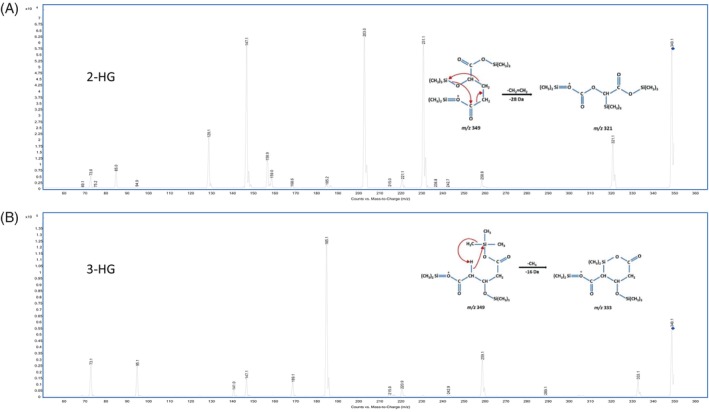
GC–MS/MS product ion mass spectra and proposed fragmentation mechanisms of tri‐TMS derivatives of (A) 2‐HG and (B) 3‐HG.

**FIGURE 3:**
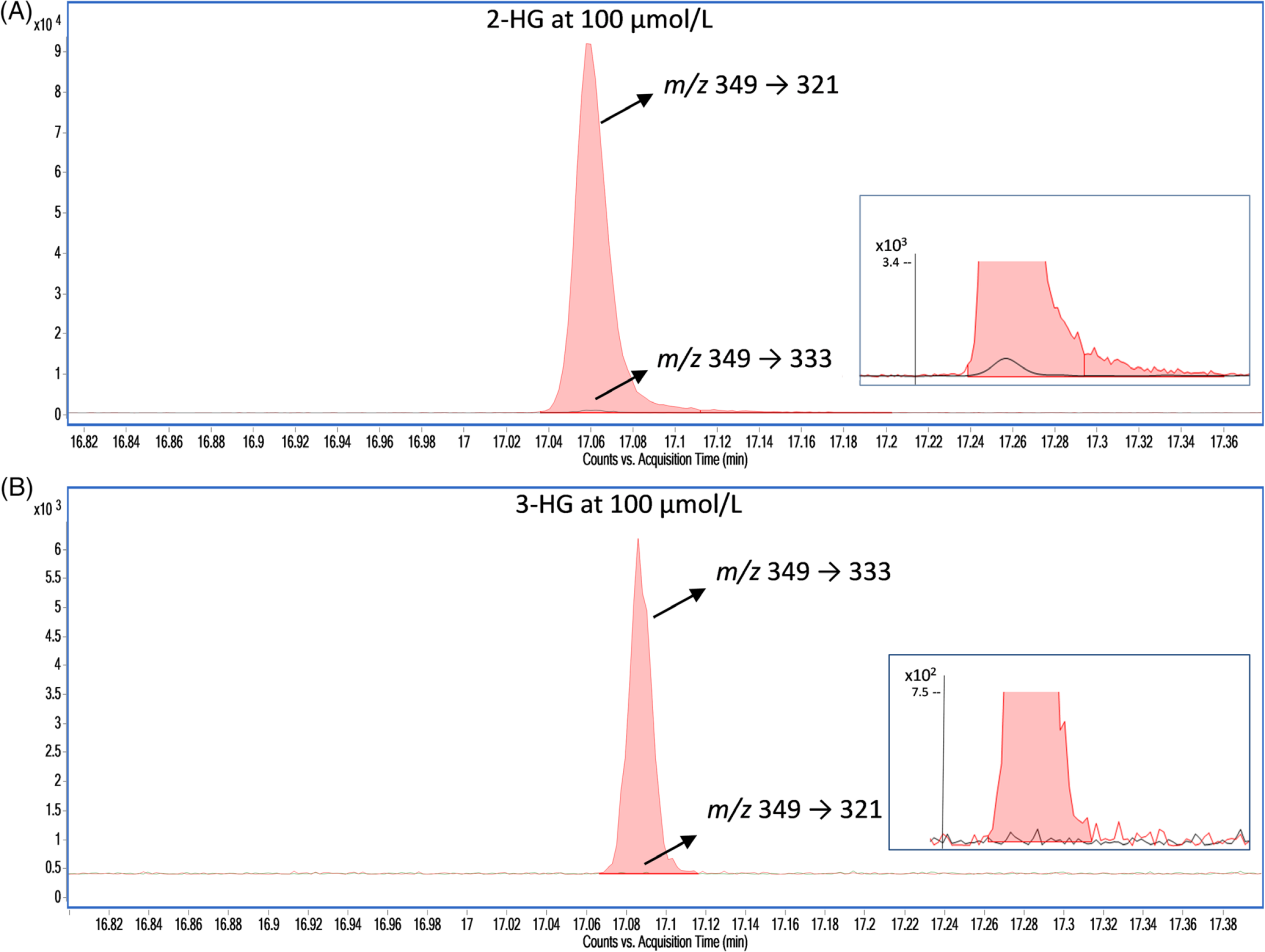
Analytical specificity of *m*/*z* 349 → 333 and *m*/*z* 349 → 321 demonstrated by monitoring the transitions in concentrated solutions of (A) 2‐HG and (B) 3‐HG. The inset boxes show an enlarged close‐up view of the baseline.

Figure 1 shows the chromatographic separations obtained using this GC–MS/MS method. The co‐elution of 2‐HG and 3‐HG is clearly visible in Figure 1B. While such overlap causes problems in routine GC–MS urine organic acid analysis, it could be tolerated in our GC–MS/MS method without compromising the accurate quantification of either molecule. This was possible by exploiting the advantages of tandem mass spectrometry in MRM mode, which has great potential for the enhancement of sensitivity and specificity.^14^ During the first stage of method development, 2‐HG and 3‐HG were analyzed separately in scan mode and the tandem mass spectrometry parameters were refined using the MassHunter Optimizer for GC/TQ (https://www.agilent.com). This software identified precursor ions and fragment ions, optimized collision energies and found the best MRM parameters. Further experiments were also conducted using different mass spectrometric parameters (collision cell energy, mass resolution, gain, dwell time, and source temperature) to find the most specific ion transitions. Figure 2 shows the fragment ion mass spectra and molecular structures of tri‐TMS‐derivatized 2‐HG (Figure 2A) and 3‐HG (Figure 2B). During their ionization process, both 2‐HG and 3‐HG generate ions corresponding to *m*/*z* 349 after the loss of a methyl radical (−15 Da).^15^ Following collision‐induced fragmentation, the *m*/*z* 349 ion of 3‐HG generates a characteristic fragment at *m*/*z* 333, corresponding to the neutral loss of methane (−16 Da),^15^ whereas this fragment is produced at extremely low abundance from 2‐HG. In contrast, the *m*/*z* 349 ion of 2‐HG produces a characteristic fragment at *m*/*z* 321, which can be attributed to the neutral loss of ethylene (−28 Da). A concerted fragmentation mechanism is proposed in Figure 2 to explain the formation of the *m*/*z* 321 ion from 2‐HG. A loss of carbon monoxide (CO) was not considered plausible to explain the m/z 321 fragment of 2‐HG, since this loss would also have been observed for 3‐HG which has the same carboxylic acid groups as 2‐HG. However, in the case of 3‐HG, the absence of two consecutive methylene groups in its structure prevents the loss of the ethylene group.

Thus, the use of the specific transitions *m*/*z* 349 → 333 for 3‐HG and *m*/*z* 349 → 321 for 2‐HG essentially eliminated any cross‐interference. In a pure 100 μM solution of 2‐HG, the *m*/*z* 333/*m*/*z* 321 response ratio was only 0.07%, and in a pure 100 μM solution of 3‐HG, the *m*/*z* 321 is undetectable. This specificity is demonstrated in Figure 3. Qualitative examination of the *m*/*z* 349 → 333 transition in a 500 μM aqueous solution of 2‐HG shows no visible peak. Moreover, the use of the qualifier ion/quantifier ion area ratios in the quantification process added a greater degree of specificity. The qualifier *m*/*z* 349 → 185 was used because this ion is abundant in the 3‐HG mass fragmentation spectrum, which is not the case for 2‐HG (Figure 2). As the (*m*/*z* 349 → 185)/(*m*/*z* 349 → 333) mean area ratio was established experimentally to be 850, if the ratio observed for a given specimen falls outside the acceptable range (550–1150, i.e., mean ± 2SD), an interference can be suspected and further investigations initiated.

The method was validated according to standard criteria, based on ISO15189 (www.iso.org) and COFRAC guidelines.^16^ Results are summarized in Table 1. The evaluated parameters included limit of quantification (LOQ), linearity, measurement range, precision, accuracy and recovery. The linearity was assessed by preparing the calibration curve in aqueous solution at concentrations of 2.5, 10, 20, 100, 200, and 400 μM of 2‐HG and GA, and 1, 2.5, 5, 25, 50, and 100 μM of 3‐HG. Successive dilutions of aqueous solutions, 1000 μM for GA and 2‐HG, 500 μM for 3‐HG, were analyzed and the lowest calculated concentrations with a relative error <20% were used to establish the lower limits of the measurement range (LOQ). Upper measurement limits were not formally determined, as they were above the highest concentrations tested. Any specimen with 3‐HG > 500 μM, or GA or 2‐HG > 1000 μM, is therefore re‐analyzed following appropriate dilution. Quality control (QC) samples in urine matrices at low and medium/high levels were used to analyze the intra‐ and inter‐day precisions. The observed coefficients of variation were all <15%. Recovery was assessed by quantifying urine fortified with known amounts of 3‐HG, 2‐HG, and GA at low, medium, and high concentrations. Accuracy was evaluated by participation in a quantitative external quality program provided by ERNDIM (the European Research Network for evaluation and improvement of screening, Diagnosis and treatment of Inherited disorders of Metabolism; www.erndim.org). The ERNDIM quantitative organic acids scheme includes eight urine samples per year, spiked at four different concentration levels. Accuracy was defined by comparing the mean quantitative results for the eight samples in our laboratory with the means of all participating laboratories (https://www.erndim.org/eqa-schemes/eqa-scheme-annual-reports/) and was acceptable according to the criteria used by the scheme organizers. Finally, diagnostic performance of the GC–MS/MS assay was further validated by analyzing urine specimens from confirmed patients. Table 2 shows GA, 3‐HG, and 2‐HG results obtained for patients with a confirmed diagnosis of GA1 (patients 1–10). 3‐HG was above the age‐related reference range in all samples analyzed from these patients, including samples from low‐excretors (e.g., patient 3, showing only a mild elevation of GA at the time of diagnosis). 3‐HG remained above the age‐related reference range in all specimens collected during follow‐up of GA1 patients on treatment, even when glutaric acid was within reference range (patients 6 and 7). Patients 11–13 do not have GA1 but are included for comparison. Patient 11 has a confirmed diagnosis of MADD (mild form), reflected by a higher level of 2‐HG compared to 3‐HG. Patients 12 and 13 both have confirmed 2‐hydroxyglutaric acidurias; their specimens show markedly elevated 2‐HG with normal 3‐HG and normal GA.

**TABLE 1:** Summary of method validation results.

Criteria	3‐HG	2‐HG	GA
Limit of quantification (LOQ)	1 μmol/L	5 μmol/L	6 μmol/L
Linearity (*r* ^2^)—0 to 400 μmol/L (3‐HG 0–100 μmol/L)	0.996	0.992	0.995
Measurement range limits	1–500 μmol/L^a^	5–1000 μmol/L^a^	6–1000 μmol/L^a^
Precision (intra‐batch)^b^			
Low value	13.3%	6.4%	3.5%
High value	5.5%	4.2%	5.3%
Precision (inter‐day)^c^			
Low value	10.2%	13.7%	14.0%
High value	14.3%	13.8%	14.5%
Accuracy	84%	85%	97%
Recovery^d^			
Low value	85%	97%	82%
Medium value	91%	113%	102%
High value	97%	116%	116%

*Note*: Accuracy was evaluated using samples from a quantitative external quality program (ERNDIM).

^a^ Highest tested concentration.

^b^ Low value 3 μM (3‐HG), 20 μM (2‐HG), 10 μM (GA). High value 200 μM for all three acids. Results expressed as relative standard deviation (RSD%).

^c^ Low value 5 μM (3‐HG), 27 μM (2‐HG), 14 μM (GA). High value 124 μM (3‐HG), 190 μM (2‐HG), 216 μM (GA). Results expressed as relative standard deviation (RSD%).

^d^ Low value 5 μM (3‐HG), 10 μM (2‐HG and GA). Median value 40 μM (3‐HG), 80 μM (2‐HG and GA). High value 100 μM (3‐HG), 200 μM (2‐HG and GA).

**TABLE 2:** Concentrations of 3‐hydroxyglutaric acid, 2‐hydroxyglutaric acid, and glutaric acid observed in urine specimens from patients with a confirmed diagnosis.

Source of urine sample	Diagnosis and context	Age at sample collection	3‐HG	2‐HG	GA
			mmol/mol creatinine	mmol/mol creatinine	mmol/mol creatinine
Patient 1	GA1—at diagnosis^a^	10 days	69.1 (<7.5)	37.9 (<53.8)	340 (<13.5)
	GA1—follow‐up^b^	20 days	46.0 (<7.5)	44.4 (<53.8)	40.8 (<13.5)
	GA1—follow‐up^b^	5 months	58.0 (<6.5)	30.1 (<29.7)	41.7 (<15.9)
	GA1—follow‐up^b^	12 months	32.3 (<6.5)	29.9 (<29.7)	47.1 (<15.9)
Patient 2	GA1—at diagnosis^a^	15 days	169 (<7.5)	30.8 (<53.8)	4023 (<13.5)
Patient 3	GA1—at diagnosis^a^	12 days	43.9 (<7.5)	35.2 (<53.8)	30.8 (<13.5)
Patient 4	GA1—at diagnosis^a^, ^c^	21 years	22.1 (<2.6)	9.8 (<6.8)	57.0 (<2.6)
Patient 5	GA1—follow‐up^b^	25 years	19.6 (<2.6)	8.6 (<6.8)	161 (<2.6)
Patient 6	GA1—follow‐up^b^	5 years	10.6 (<6.4)	12.8 (<15.6)	1.7 (<3.8)
Patient 7	GA1—follow‐up^b^	17 months	50.5 (<6.5)	10.9 (<29.7)	15.0 (<15.9)
Patient 8	GA1—at diagnosis^a^	2 months	294 (<6.5)	85.0 (<29.7)	7023 (<15.9)
Patient 9	GA1—at diagnosis^a^	4 months	611 (<6.5)	56.8 (<29.7)	7308 (<15.9)
Patient 10	GA1—follow‐up^b^	12 years	75.5 (<2.6)	9.4 (<6.8)	2950 (<2.6)
Patient 11	MADD—follow‐up^b^	16 years	3.0 (<2.6)	23.8 (<6.8)	1.0 (<2.6)
Patient 12	d‐2‐Hydroxyglutaric aciduria^d^	5 years	5.8 (<6.4)	638 (<15.6)	3.2 (<3.8)
Patient 13	2‐Hydroxyglutaric aciduria^d^	7 years	3.9 (<6.4)	1641 (<15.6)	1.3 (<3.8)

*Note*: Values less than 100 are shown to 1 decimal place. Laboratory reference values are shown in parentheses for each result. These reflect 95th percentile values of controls in each of the following age groups: 0–2 months; 2 months to 2 years; 2 years to 12 years; >12 years.

Abbreviations: GA, glutaric acid; GA1, glutaric aciduria type 1; 3‐HG, 3‐hydroxyglutaric acid; 2‐HG, 2‐hydroxyglutaric acid; MADD, multiple acyl‐CoA dehydrogenase deficiency.

^a^ First specimen received for diagnostic testing; presumably before initiation of any treatment.

^b^ Follow‐up specimen received for “monitoring,” in a context of treatment or management.

^c^ Incidental diagnosis of “maternal GA1,” following a positive newborn screen of an unaffected infant.

^d^ Specimen received from external proficiency scheme, presumably collected during patient follow‐up.

## 4 DISCUSSION

Accurate identification of urinary 3‐HG is important for diagnosing GA1, but can be challenging in routine GC–MS profile analysis, due to co‐elution and spectral similarity with its isomer 2‐HG.^10^ We therefore focused on increasing the analytical specificity for 3‐HG in this new GC–MS/MS assay. The method presented here represents an enhancement of a GC–MS method previously established in our laboratory. Now, the use of GC–MS/MS with MRM gives higher selectivity, resulting in less interference of co‐eluting compounds, better signal‐to‐noise ratios and more reliable identification of 3‐HG.

These advantages allow the use of two more specific ion transitions produced during the tandem mass spectrometric fragmentation of the trimethylsilyl (TMS) derivatives of 2‐HG and 3‐HG respectively. The loss of a CH_2_=CH_2_ group on the 2‐HG precursor ion led to a molecular rearrangement yielding the *m*/*z* 321 ion, which is not produced during the 3‐HG fragmentation process. Conversely, fragmentation of the 3‐HG precursor ion produced the *m*/*z* 333 fragment, which is only generated at very low abundance from 2‐HG. Results of method validation show good analytical performance.

This GC–MS/MS method has now been in routine use in our clinical laboratory for over a year. It is a key step in the process of confirmation (or refutation) of a diagnosis of GA1 in “screen positive” infants referred by NBS programs serving several Canadian provinces. This test is also used to help confirm or rule out a diagnosis of GA1 in contexts of clinical suspicion, sometimes being requested specifically to clarify ambiguous results obtained from urine organic acid profile analysis by GC–MS in other laboratories.

To the best of our knowledge, this is the first report of a stable isotope dilution GC–MS/MS method designed to differentiate 3‐HG from 2‐HG and thus to facilitate the quantitation of 3‐HG. A method using liquid chromatography–tandem mass spectrometry (LC–MS/MS) to quantitate 3‐HG in urine, dried on filter paper, was published in 2011.^17^ It involved derivatization with a novel reagent, which was synthesized in‐house. Although this approach offered advantages over existing GC–MS methods, it appears not to have been widely adopted in other laboratories, perhaps reflecting a lack of commercial availability of the derivatization reagent and reluctance to perform its synthesis. In contrast, the trimethylsilyl derivatization step included in our GC–MS/MS method has long been widely used in urine organic acid analysis by GC–MS.^5^ While GC–MS/MS technology is not yet implanted in many clinical laboratories, there is increasing interest and its gradual adoption is likely, given the anticipated benefits.

In summary, gas chromatography‐tandem mass spectrometry (GC–MS/MS) allows clear identification and accurate quantification of 3‐HG, despite co‐elution of 2‐HG or of other substances which can potentially interfere in the usual GC–MS methods. This selectivity improvement is particularly useful for follow‐up testing of infants with elevated glutarylcarnitine on NBS which raises a possibility of GA1. The method is also well suited for diagnosis of GA1 in the clinical laboratory, especially for low excretor patients (Table 2). We have established and validated a simple, sensitive and specific GC–MS/MS isotope dilution assay to quantify 3‐HG, 2‐HG, and GA, which serves as an example demonstrating the capability and advantages of using tandem mass spectrometry for diagnostic testing in clinical biochemical genetics laboratories.

## AUTHOR CONTRIBUTIONS

Denis Cyr performed the method development and validation and wrote the first draft of the manuscript. Michel Boutin was involved in the description of chemical mechanisms and contributed to revision of the manuscript. Bruno Maranda reviewed clinical and scientific aspects of the manuscript. Paula J Waters contributed to the conception of this study, literature review, and critical review and editing of the manuscript. All authors reviewed the final manuscript and gave approval for submission.

## CONFLICT OF INTEREST STATEMENT

Denis Cyr, Michel Boutin, Bruno Maranda, and Paula J Waters declare that they have no conflict of interest.

## ETHICS STATEMENT

Ethics approval was not required for this study, which was performed in accordance with standard practices for method development and validation within a clinical diagnostic laboratory.

## INFORMED CONSENT AND ANIMAL RIGHTS

This article does not contain any studies with human or animal subjects performed by any of the authors. All specimens analyzed were originally submitted for clinical laboratory testing. This article does not contain information identifying individual patients or clinical description of individual patients.

## Data Availability

The data, tools, and material (or their source) that support the results and conclusions presented in this article are available from the corresponding authors upon reasonable request.
